# *N*-(4,4′-Dibromo-[1,1′-biphen­yl]-2-yl)benzamide

**DOI:** 10.1107/S1600536813000597

**Published:** 2013-01-12

**Authors:** J. Josephine Novina, G. Vasuki, Abhishek Baheti, K. R. Justin Thomas

**Affiliations:** a Department of Physics, Idhaya College for Women, Kumbakonam-1, India; b Department of Physics, Kunthavai Naachiar Govt. Arts College (W) (Autonomous), Thanjavur-7, India; c Organic Materials Lab, Department of Chemistry, Indian Institute of Technology Roorkee, Roorkee 247 667, India

## Abstract

In the title compound, C_19_H_13_Br_2_NO, the dihedral angle between the rings of the biphenyl group is 53.59 (14)°. The ring of the benzamide group is inclined to the phenyl rings of the biphenyl group by 23.87 (15) and 75.89 (15)°. There are no significant inter­molecular inter­actions in the crystal structure.

## Related literature

For applications of the title compound, see: Libman & Slack (1951 ▶); Mandadapu *et al.* (2009 ▶); Youn & Bihn (2009 ▶); Yulan *et al.* (2010 ▶). For pharmacological properties of biphenyl aniline, see: Zhu *et al.* (2008 ▶). For related structures, see: Li & Cui (2011 ▶); Kuś *et al.* (2009 ▶); Hammond *et al.* (2009 ▶); Gowda *et al.* (2010 ▶); Novina *et al.* (2012 ▶).
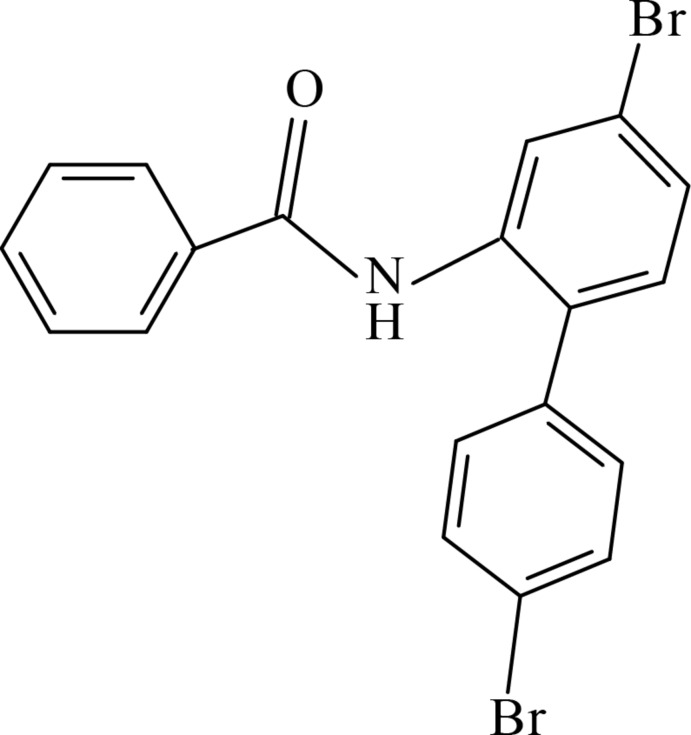


## Experimental

### 

#### Crystal data

C_19_H_13_Br_2_NO
*M* *_r_* = 431.12
Monoclinic, 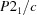

*a* = 9.0188 (5) Å
*b* = 11.6415 (9) Å
*c* = 16.0068 (12) Å
β = 100.737 (2)°
*V* = 1651.2 (2) Å^3^
*Z* = 4
Mo *K*α radiation
μ = 4.91 mm^−1^
*T* = 293 K
0.30 × 0.25 × 0.20 mm

#### Data collection

Bruker Kappa APEXII CCD diffractometer
Absorption correction: multi-scan (*SADABS*; Bruker, 2004 ▶) *T*_min_ = 0.238, *T*_max_ = 0.374
16420 measured reflections
3453 independent reflections
2302 reflections with *I* > 2σ(*I*)
*R*_int_ = 0.032

#### Refinement

*R*[*F* ^2^ > 2σ(*F* ^2^)] = 0.033
*wR*(*F* ^2^) = 0.075
*S* = 1.01
3453 reflections
208 parameters
H-atom parameters constrained
Δρ_max_ = 0.46 e Å^−3^
Δρ_min_ = −0.49 e Å^−3^

### 

Data collection: *APEX2* (Bruker, 2004 ▶); cell refinement: *APEX2* and *SAINT* (Bruker, 2004 ▶); data reduction: *SAINT* and *XPREP* (Bruker, 2004 ▶); program(s) used to solve structure: *SHELXS97* (Sheldrick, 2008 ▶); program(s) used to refine structure: *SHELXL97* (Sheldrick, 2008 ▶); molecular graphics: *ORTEP-3* (Farrugia, 2012 ▶); software used to prepare material for publication: *PLATON* (Spek, 2009 ▶).

## Supplementary Material

Crystal structure: contains datablock(s) I, global. DOI: 10.1107/S1600536813000597/su2551sup1.cif

Structure factors: contains datablock(s) I. DOI: 10.1107/S1600536813000597/su2551Isup2.hkl

Supplementary material file. DOI: 10.1107/S1600536813000597/su2551Isup3.cml

Additional supplementary materials:  crystallographic information; 3D view; checkCIF report

## Figures and Tables

**Fig. 1.**
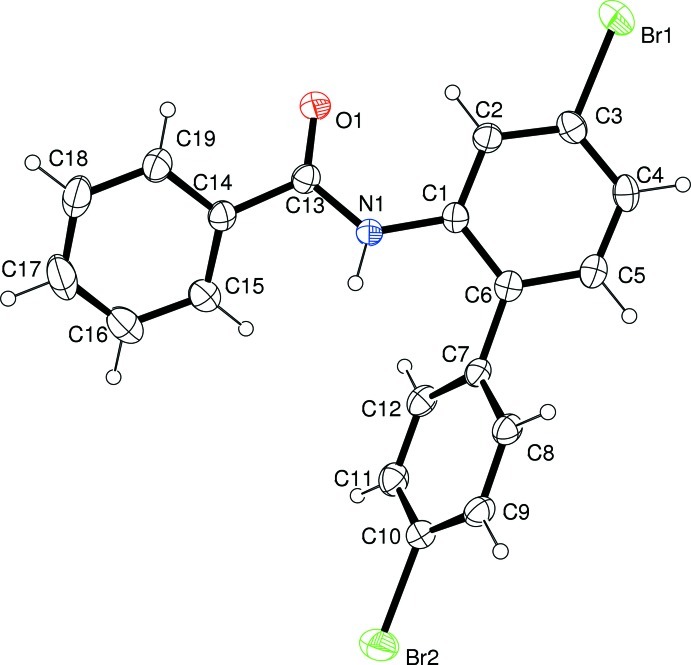
The molecular structure of the title molecule, with the atom labelling. Displacement ellipsoids are drawn at the 50% probability level.

**Fig. 2.**
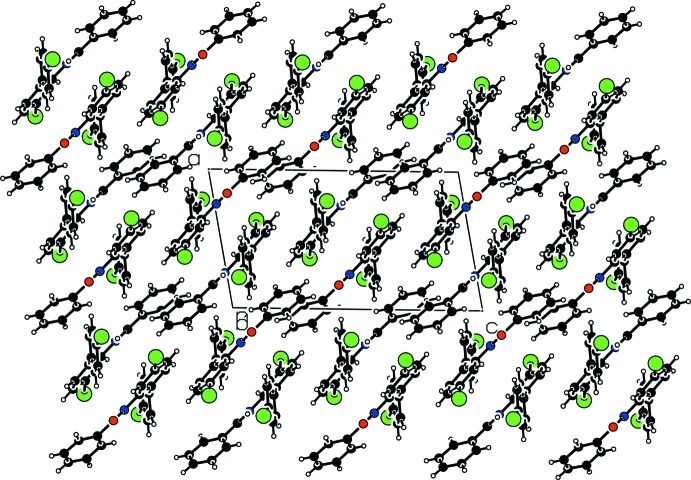
Crystal packing of the title compound viewed along the b axis.

## supplementary crystallographic information

### Comment

Biphenyl aniline, a subclass of biaryl compounds, has been recognized as a privileged structure in drug discovery. Its derivatives have been pursued as anti–phlogistic, analgesic, anti–obesity, and anti–tumor agents (Zhu *et al.*, 2008). Amide substituted biphenyl derivatives are commonly used to develop antiparasitic agents for the treatment of African sleeping sickness disease (Libman & Slack, 1951; Mandadapu *et al.*, 2009; Youn & Bihn, 2009; Yulan *et al.*, 2010). Benzamides are recognized as one of the important bioactive skeletons and exhibit various potent pharmaceutical activities. As part of our studies on the substituent effects on the structures and other aspects of dibromo biphenyl derivatives, 4,4'-Dibromo-2-nitrobiphenyl (Novina *et al.*, 2012), in the present work we report herein on the synthesis and crystal structure of the title compound.

In the molecular structure of the title compound (Fig. 1), the two benzene rings of the biphenyl group are twisted with respect to each other by 53.59 (14)°, which is similar to to the arrangement [53.52 (14)°] found in 2,5-Bis(bromomethyl)biphenyl (Kuś *et al.*, 2009), but is somewhat larger than the angle of 45.5 (2)° found in 3,3',5,5'-Tetranitrobiphenyl (Hammond *et al.*, 2009). The amide unit C1—N1—C13(O1)—C14 is planar [r.m.s deviation = 0.013 Å], and subtends dihedral angles of 12.38 (12)° and 32.34 (11)° respectively to the C1-C6 and C14-C19 phenyl rings. These two aromatic rings are inclined to one another by 23.87 (15)°, while rings C7-C12 and C14-C19 are inclined to one another by 75.89 (15)°. The C13═O1 and C13—N1 bond distances are 1.221 (3) and 1.360 (3) Å, respectively, showing the electron delocalization in the amide fragment. The N—H and C═O bonds in the amide group are *anti* to each other, similar to that observed in 2-Chloro-*N*-(2,3-dimethylphenyl)-benzamide (Gowda *et al.*, 2010), and *N*-(3,5-Dimethoxyphenyl)benzamide (Li & Cui, 2011). The length of the bond connecting the phenyl rings, 1.489 (4) Å, is close to the standard value of 1.48 Å for a C*sp*^2^—C*sp*^2^ single bond.

In the crystal, there are no significant interactions and the structure is stabilized by Van der Waals interactions (Fig. 2).

### Experimental

To a dry THF solution of 4,4'-dibromo-[1,1'-biphenyl]-2-amine (3.27 g, 10 mmol) and triethylamine (3 ml) was added drop wise a dry THF solution (40 ml) of benzoyl chloride at 273 K. After stirring at room temperature for 20 h, the solution was poured into water (80 ml) and extracted with dichloromethane (2 × 50 ml). The combined organic extracts were dried over anhydrous Na_2_SO_4_ and evaporated to dryness. This gave white solid which was further recrystallized with dichloromethane-hexanes [Yield 3.0 g (70%); M.p. 443–445 K]. HRMS calcd. for C_19_H_13_Br_2_NO [*M*]^+^ m/*z* 428.9364 found 428.9363. Spectroscopic data for the title compound is available in the archived CIF.

### Refinement

H atoms were placed in geometrically idealized positions and constrained to ride on their parent atoms: N—H = 0.86 Å and C—H = 0.93 Å with *U*_iso_(H) = 1.2*U*_eq_(C,*N*).

### Crystal data

**Table:**

C_19_H_13_Br_2_NO	*F*(000) = 848
*M**_r_* = 431.12	*D*_x_ = 1.734 Mg m^−^^3^
Monoclinic, *P*2_1_/*c*	Mo *K*α radiation, λ = 0.71073 Å
Hall symbol: -P 2ybc	Cell parameters from 3453 reflections
*a* = 9.0188 (5) Å	θ = 2.2–26.6°
*b* = 11.6415 (9) Å	µ = 4.91 mm^−^^1^
*c* = 16.0068 (12) Å	*T* = 293 K
β = 100.737 (2)°	Block, colourless
*V* = 1651.2 (2) Å^3^	0.30 × 0.25 × 0.20 mm
*Z* = 4	

### Data collection

**Table:**

Bruker Kappa APEXII CCD diffractometer	3453 independent reflections
Radiation source: fine-focus sealed tube	2302 reflections with *I* > 2σ(*I*)
Graphite monochromator	*R*_int_ = 0.032
ω and φ scan	θ_max_ = 26.6°, θ_min_ = 2.2°
Absorption correction: multi-scan (*SADABS*; Bruker, 2004)	*h* = −11→11
*T*_min_ = 0.238, *T*_max_ = 0.374	*k* = −14→14
16420 measured reflections	*l* = −19→20

### Refinement

**Table:**

Refinement on *F*^2^	Primary atom site location: structure-invariant direct methods
Least-squares matrix: full	Secondary atom site location: difference Fourier map
*R*[*F*^2^ > 2σ(*F*^2^)] = 0.033	Hydrogen site location: inferred from neighbouring sites
*wR*(*F*^2^) = 0.075	H-atom parameters constrained
*S* = 1.01	*w* = 1/[σ^2^(*F*_o_^2^) + (0.0267*P*)^2^ + 1.1775*P*] where *P* = (*F*_o_^2^ + 2*F*_c_^2^)/3
3453 reflections	(Δ/σ)_max_ = 0.001
208 parameters	Δρ_max_ = 0.46 e Å^−^^3^
0 restraints	Δρ_min_ = −0.49 e Å^−^^3^

### Special details

**Table:**

Experimental. Spectroscopic data for the title compound: IR (ν_N—H_) 3399 cm^-1^, 1567 cm^-1^, (ν_C__═__O_) 1666 cm^-1^, ^1^H NMR (CDCl_3_, 500 MHz) δ: 8.74 (d, J = 1.5 Hz, 1 H), 7.85 (s, 1H), 7.60 (dd, J = 6.5, 2.0 Hz, 2 H), 7.59–7.61 (m, 2 H), 7.52–7.55 (m, 1 H), 7.42–7.45 (m, 2 H), 7.35 (d, J = 1.5 Hz, 1 H), 7.30 (dd, J = 6.5, 2.0 Hz, 2 H), 7.11 (d, J = 8.0 Hz, 1 H); ^13^C NMR (CDCl_3_, 125 MHz) δ 165.1, 136.0, 135.8, 134.1, 133.7, 32.6, 131.1, 130.8, 130.2, 130.1, 129.0, 128.5, 127.7, 126.8, 124.6, 122.9, 122.7.
Geometry. All e.s.d.'s (except the e.s.d. in the dihedral angle between two l.s. planes) are estimated using the full covariance matrix. The cell e.s.d.'s are taken into account individually in the estimation of e.s.d.'s in distances, angles and torsion angles; correlations between e.s.d.'s in cell parameters are only used when they are defined by crystal symmetry. An approximate (isotropic) treatment of cell e.s.d.'s is used for estimating e.s.d.'s involving l.s. planes.
Refinement. Refinement of *F*^2^ against ALL reflections. The weighted *R*-factor *wR* and goodness of fit *S* are based on *F*^2^, conventional *R*-factors *R* are based on *F*, with *F* set to zero for negative *F*^2^. The threshold expression of *F*^2^ > σ(*F*^2^) is used only for calculating *R*-factors(gt) *etc*. and is not relevant to the choice of reflections for refinement. *R*-factors based on *F*^2^ are statistically about twice as large as those based on *F*, and *R*- factors based on ALL data will be even larger.

### Fractional atomic coordinates and isotropic or equivalent isotropic displacement parameters (Å^2^)

**Table:**

	*x*	*y*	*z*	*U*_iso_*/*U*_eq_	
Br1	0.63621 (4)	0.21302 (3)	0.15668 (2)	0.06302 (13)	
Br2	0.22124 (4)	1.08134 (3)	0.04697 (3)	0.07108 (15)	
O1	0.1733 (2)	0.33800 (17)	−0.05842 (14)	0.0542 (6)	
C1	0.3653 (3)	0.4886 (2)	0.06373 (17)	0.0342 (6)	
C8	0.4769 (3)	0.7869 (2)	0.09646 (18)	0.0414 (7)	
H8	0.5785	0.7681	0.1022	0.050*	
C19	0.0269 (3)	0.4448 (3)	−0.2115 (2)	0.0496 (8)	
H19	0.0688	0.3731	−0.2180	0.060*	
N1	0.2512 (2)	0.51464 (19)	−0.00712 (14)	0.0380 (6)	
H1	0.2333	0.5866	−0.0160	0.046*	
C11	0.1763 (3)	0.8444 (3)	0.07935 (19)	0.0480 (8)	
H11	0.0750	0.8640	0.0737	0.058*	
C2	0.4251 (3)	0.3787 (2)	0.07720 (18)	0.0397 (7)	
H2	0.3859	0.3185	0.0417	0.048*	
C4	0.6025 (3)	0.4467 (3)	0.19891 (18)	0.0455 (8)	
H4	0.6806	0.4320	0.2444	0.055*	
C3	0.5430 (3)	0.3604 (3)	0.14369 (18)	0.0410 (7)	
C7	0.3728 (3)	0.7005 (2)	0.10278 (17)	0.0361 (6)	
C12	0.2218 (3)	0.7322 (3)	0.09524 (19)	0.0440 (7)	
H12	0.1504	0.6766	0.1010	0.053*	
C14	0.0602 (3)	0.5010 (2)	−0.13340 (18)	0.0372 (7)	
C9	0.4329 (3)	0.8994 (2)	0.08194 (19)	0.0439 (7)	
H9	0.5043	0.9562	0.0789	0.053*	
C10	0.2825 (3)	0.9271 (2)	0.07198 (19)	0.0437 (7)	
C5	0.5431 (3)	0.5548 (3)	0.18479 (19)	0.0455 (7)	
H5	0.5831	0.6139	0.2213	0.055*	
C6	0.4249 (3)	0.5796 (2)	0.11777 (17)	0.0367 (6)	
C13	0.1656 (3)	0.4425 (2)	−0.06326 (18)	0.0373 (7)	
C18	−0.0677 (4)	0.4950 (3)	−0.2791 (2)	0.0626 (10)	
H18	−0.0875	0.4581	−0.3315	0.075*	
C17	−0.1326 (4)	0.5993 (4)	−0.2690 (3)	0.0746 (11)	
H17	−0.1956	0.6334	−0.3149	0.089*	
C15	−0.0075 (3)	0.6061 (3)	−0.1242 (2)	0.0493 (8)	
H15	0.0130	0.6446	−0.0725	0.059*	
C16	−0.1055 (4)	0.6535 (3)	−0.1922 (3)	0.0664 (10)	
H16	−0.1532	0.7228	−0.1855	0.080*	

### Atomic displacement parameters (Å^2^)

**Table:**

	*U*^11^	*U*^22^	*U*^33^	*U*^12^	*U*^13^	*U*^23^
Br1	0.0675 (2)	0.0512 (2)	0.0659 (2)	0.01286 (17)	0.00079 (18)	0.01402 (17)
Br2	0.0641 (2)	0.0444 (2)	0.0985 (3)	0.00851 (17)	−0.0009 (2)	0.0004 (2)
O1	0.0578 (13)	0.0335 (12)	0.0629 (14)	−0.0026 (10)	−0.0104 (11)	−0.0010 (10)
C1	0.0326 (15)	0.0391 (16)	0.0314 (15)	−0.0046 (12)	0.0071 (12)	0.0051 (13)
C8	0.0324 (15)	0.0438 (18)	0.0471 (18)	−0.0032 (13)	0.0048 (13)	−0.0074 (15)
C19	0.0484 (18)	0.0491 (19)	0.0467 (19)	−0.0067 (15)	−0.0033 (15)	−0.0017 (16)
N1	0.0417 (13)	0.0296 (13)	0.0390 (14)	−0.0036 (10)	−0.0018 (11)	0.0029 (11)
C11	0.0359 (16)	0.054 (2)	0.052 (2)	0.0038 (15)	0.0044 (14)	−0.0088 (16)
C2	0.0421 (16)	0.0373 (16)	0.0389 (17)	−0.0044 (13)	0.0055 (14)	0.0031 (13)
C4	0.0430 (17)	0.060 (2)	0.0311 (16)	0.0009 (15)	0.0000 (14)	0.0086 (15)
C3	0.0417 (16)	0.0442 (17)	0.0382 (17)	0.0009 (14)	0.0104 (14)	0.0127 (14)
C7	0.0369 (15)	0.0385 (16)	0.0316 (15)	−0.0024 (13)	0.0035 (12)	−0.0055 (13)
C12	0.0369 (16)	0.0481 (19)	0.0473 (18)	−0.0097 (14)	0.0082 (14)	−0.0066 (15)
C14	0.0324 (14)	0.0391 (16)	0.0394 (17)	−0.0069 (12)	0.0050 (13)	0.0024 (13)
C9	0.0408 (17)	0.0397 (17)	0.0498 (19)	−0.0085 (13)	0.0052 (14)	−0.0060 (14)
C10	0.0478 (18)	0.0375 (17)	0.0428 (18)	0.0015 (14)	0.0003 (14)	−0.0059 (14)
C5	0.0442 (17)	0.0528 (19)	0.0368 (17)	−0.0030 (15)	0.0004 (14)	−0.0036 (15)
C6	0.0349 (15)	0.0421 (17)	0.0337 (16)	−0.0037 (13)	0.0083 (13)	0.0013 (13)
C13	0.0348 (15)	0.0381 (17)	0.0393 (17)	−0.0065 (12)	0.0072 (13)	0.0005 (13)
C18	0.063 (2)	0.073 (3)	0.045 (2)	−0.019 (2)	−0.0081 (18)	0.0018 (18)
C17	0.060 (2)	0.086 (3)	0.068 (3)	0.001 (2)	−0.015 (2)	0.026 (2)
C15	0.0505 (18)	0.0472 (19)	0.0496 (19)	0.0029 (15)	0.0079 (16)	0.0050 (15)
C16	0.062 (2)	0.060 (2)	0.074 (3)	0.0151 (18)	0.005 (2)	0.016 (2)

### Geometric parameters (Å, º)

**Table:**

Br1—C3	1.904 (3)	C4—C5	1.370 (4)
Br2—C10	1.899 (3)	C4—C3	1.379 (4)
O1—C13	1.221 (3)	C4—H4	0.9300
C1—C2	1.390 (4)	C7—C12	1.394 (4)
C1—C6	1.409 (4)	C7—C6	1.489 (4)
C1—N1	1.415 (3)	C12—H12	0.9300
C8—C9	1.376 (4)	C14—C15	1.388 (4)
C8—C7	1.393 (4)	C14—C13	1.493 (4)
C8—H8	0.9300	C9—C10	1.374 (4)
C19—C18	1.377 (4)	C9—H9	0.9300
C19—C14	1.393 (4)	C5—C6	1.395 (4)
C19—H19	0.9300	C5—H5	0.9300
N1—C13	1.360 (3)	C18—C17	1.371 (5)
N1—H1	0.8600	C18—H18	0.9300
C11—C12	1.378 (4)	C17—C16	1.362 (5)
C11—C10	1.379 (4)	C17—H17	0.9300
C11—H11	0.9300	C15—C16	1.382 (4)
C2—C3	1.374 (4)	C15—H15	0.9300
C2—H2	0.9300	C16—H16	0.9300
			
C2—C1—C6	120.3 (2)	C15—C14—C19	118.9 (3)
C2—C1—N1	121.7 (2)	C15—C14—C13	123.6 (3)
C6—C1—N1	117.9 (2)	C19—C14—C13	117.5 (3)
C9—C8—C7	121.5 (3)	C10—C9—C8	119.4 (3)
C9—C8—H8	119.2	C10—C9—H9	120.3
C7—C8—H8	119.2	C8—C9—H9	120.3
C18—C19—C14	120.3 (3)	C9—C10—C11	120.8 (3)
C18—C19—H19	119.8	C9—C10—Br2	119.2 (2)
C14—C19—H19	119.8	C11—C10—Br2	119.9 (2)
C13—N1—C1	129.5 (2)	C4—C5—C6	122.4 (3)
C13—N1—H1	115.3	C4—C5—H5	118.8
C1—N1—H1	115.3	C6—C5—H5	118.8
C12—C11—C10	119.3 (3)	C5—C6—C1	117.8 (3)
C12—C11—H11	120.3	C5—C6—C7	119.5 (3)
C10—C11—H11	120.3	C1—C6—C7	122.6 (2)
C3—C2—C1	119.0 (3)	O1—C13—N1	123.7 (3)
C3—C2—H2	120.5	O1—C13—C14	121.5 (3)
C1—C2—H2	120.5	N1—C13—C14	114.8 (2)
C5—C4—C3	118.1 (3)	C17—C18—C19	119.9 (3)
C5—C4—H4	121.0	C17—C18—H18	120.1
C3—C4—H4	121.0	C19—C18—H18	120.1
C2—C3—C4	122.4 (3)	C16—C17—C18	120.5 (3)
C2—C3—Br1	119.1 (2)	C16—C17—H17	119.7
C4—C3—Br1	118.4 (2)	C18—C17—H17	119.7
C8—C7—C12	117.6 (3)	C16—C15—C14	119.9 (3)
C8—C7—C6	119.9 (2)	C16—C15—H15	120.1
C12—C7—C6	122.5 (2)	C14—C15—H15	120.1
C11—C12—C7	121.3 (3)	C17—C16—C15	120.4 (3)
C11—C12—H12	119.4	C17—C16—H16	119.8
C7—C12—H12	119.4	C15—C16—H16	119.8
			
C2—C1—N1—C13	11.6 (4)	C4—C5—C6—C7	−175.9 (3)
C6—C1—N1—C13	−172.7 (3)	C2—C1—C6—C5	−0.8 (4)
C6—C1—C2—C3	−0.1 (4)	N1—C1—C6—C5	−176.5 (2)
N1—C1—C2—C3	175.5 (2)	C2—C1—C6—C7	175.5 (2)
C1—C2—C3—C4	1.4 (4)	N1—C1—C6—C7	−0.2 (4)
C1—C2—C3—Br1	−174.8 (2)	C8—C7—C6—C5	51.6 (4)
C5—C4—C3—C2	−1.7 (4)	C12—C7—C6—C5	−127.9 (3)
C5—C4—C3—Br1	174.6 (2)	C8—C7—C6—C1	−124.6 (3)
C9—C8—C7—C12	−1.0 (4)	C12—C7—C6—C1	55.9 (4)
C9—C8—C7—C6	179.5 (3)	C1—N1—C13—O1	2.2 (5)
C10—C11—C12—C7	−0.9 (5)	C1—N1—C13—C14	−177.1 (2)
C8—C7—C12—C11	2.1 (4)	C15—C14—C13—O1	148.3 (3)
C6—C7—C12—C11	−178.4 (3)	C19—C14—C13—O1	−30.4 (4)
C18—C19—C14—C15	2.2 (4)	C15—C14—C13—N1	−32.3 (4)
C18—C19—C14—C13	−179.0 (3)	C19—C14—C13—N1	149.0 (3)
C7—C8—C9—C10	−1.1 (5)	C14—C19—C18—C17	−1.7 (5)
C8—C9—C10—C11	2.3 (5)	C19—C18—C17—C16	−0.7 (6)
C8—C9—C10—Br2	−177.2 (2)	C19—C14—C15—C16	−0.4 (4)
C12—C11—C10—C9	−1.3 (5)	C13—C14—C15—C16	−179.1 (3)
C12—C11—C10—Br2	178.3 (2)	C18—C17—C16—C15	2.5 (6)
C3—C4—C5—C6	0.7 (4)	C14—C15—C16—C17	−2.0 (5)
C4—C5—C6—C1	0.5 (4)		
